# Brief research report: Fertility, teat, and body condition of foster cows in a cow–calf contact system

**DOI:** 10.3389/fvets.2025.1678081

**Published:** 2026-01-21

**Authors:** Katharina A. Zipp, Rebecca Franz-Wippermann, Ute Knierim

**Affiliations:** Farm Animal Behaviour and Husbandry, University of Kassel, Witzenhausen, Germany

**Keywords:** animal welfare, body condition score (BCS), calf rearing, cow–calf contact, dairy cow, milking, nursing, udder health

## Abstract

One alternative to early cow–calf separation is continuous foster cow-calf contact, where one cow nurses two to four calves without being milked. However, multiple sucklings may compromise teat and body condition and affect fertility. Therefore, the prevalence of dry teats, teat lesions, abnormal body condition scores (BCS >3.75 or <2.5), rapid BCS changes (>0.5 absolute range), calving intervals, and number of inseminations to conception were compared between 18 foster cows kept in two groups of 11–12 cows with 46–48 calves and 18 milked cows in a commercial Holstein-Friesian herd. Four scorings were conducted in approximately 4-week intervals from weeks 2 to 16 postpartum. The final scoring was performed during weaning in one foster group and after weaning in the other. Associations between foster cows’ BCS and teat lesions were further analyzed. Teat lesion prevalence was significantly higher in foster cows between weeks 10 and 14 postpartum, but not during or after weaning, indicating increasing calf independence from milk. Given that teat lesions may cause pain, increase infection risk, and reflect negative cow–calf interactions, they represent a welfare concern. No significant differences were found in teat dryness, overall body condition, or fertility outcomes. However, foster cows showed a trend toward overconditioning compared to milking cows (BCS > 3.75) from weeks 6 to 16, and foster cows with lesions had a numerically higher BCS compared to foster cows without teat lesions (medium effect size). These results suggest that large-scale studies are needed to investigate the causes of teat lesions, particularly in relation to individual cow acceptance of multiple suckling in foster systems.

## 1 Introduction

Early cow–calf separation remains standard on dairy farms, yet it faces increasing societal criticism (1, 2). In response, there is growing interest among farmers in cow–calf contact (CCC) systems, in which calves are nursed by their dams for several weeks to months—often alongside regular milking (3–5). However, farmers report impaired milk ejection during the milking of dams with calf contact, which can be economically relevant (6, 7). Furthermore, building constraints may present technical or logistical challenges (6). Therefore, some farmers prefer to allow only some cows from their herd for calf contact, i.e., foster CCC (6, 8, 9). Foster cows typically have permanent contact with two to four calves in a dedicated area of the farm or on pasture. They are not milked during the nursing period. The cow’s own calf may be among those nursed. The other alien calves are called foster calves [reviewed by (10, 11)].

From an animal welfare perspective, older experimental studies have reported poorer teat skin condition in foster cows nursing two to four calves compared to cows that were only milked (12, 13). Rasmussen and Larsen (14) compared udder halves per cow that were suckled with udder halves that were milked. They found worse teat skin in the suckled udder halves, although the sample size of only four cows indicates that the results should be interpreted with caution. In contrast, a recent on-farm assessment in Switzerland found no differences in teat condition between cows with whole-day or twice-day contact with their own calves and those that were only milked (7). Possibly, disturbances during suckling can increase the risk of teat bites by calves. Franz-Wippermann et al. (15) observed in a foster CCC system that cows kicked during more suckling attempts by foster calves than of their own calves. The median number of 25 suckling bouts per 24 h was high for both foster and own calves, pointing to disturbances during suckling (15). A pilot study without a control group assessed twice as many teat lesions in foster cows at the end of the nursing period of 3.5 months postpartum (p.p.) compared to the start of lactation (16). Moreover, since foster cows’ teats are typically not dipped to prevent calves from ingesting the disinfectant (17), the absence of the moisturizing agent may lead to dry teat skin.

The number of calves per foster cow in relation to her milk yield and acceptance of suckling influences whether and how frequently the foster cow’s udder is emptied (17). In several studies, an effect of udder emptying frequency on cow’s body weight or BCS has been found: An increase in milk removal frequency from twice daily to more often through milking alone or in combination with suckling led to greater weight loss (18) or decreasing BCS in dairy cows (19–21). Milking once per day resulted in an increasing BCS compared to a decreasing BCS when milked twice or thrice (22). A low BCS reflects insufficient nutrient intake, which may lead to delayed first ovulation after calving and decreased pregnancy rates [reviewed by (23)]. In addition, prolonged postpartum anestrus (for more than 90 days) can be associated with an existing cow–calf bond (23). Although this phenomenon can be present in CCC [reviewed by (24)], Zipp and Knierim (25) found no differences in calving intervals between whole-day and half-day contact dams and cows in the non-nursing group. A reason may be a shorter calving-conception interval in dams [reviewed by (10)]. As CCC lasted only 9 weeks in the study of Zipp and Knierim (25), it remains unclear if foster CCC contact over a longer period might negatively impact fertility.

More recent investigations on foster cow systems are lacking. Therefore, the objective of the current study was to test the following hypotheses: (1) teat lesions and dry teat skin are more prevalent in foster cows than in cows that are milked; (2) the percentage of overconditioned cows (BCS > 3.75) is lower, and the percentage of underconditioned cows (BCS < 2.5) is higher in foster cows; additionally they experience more rapid changes in body condition (BCS range >0.5 over the study period); (3) as rejection of suckling attempts by calves may lead to biting as well as less milk removal, foster cows with teat lesions have a higher BCS than foster cows without teat lesions; (4) foster cows that nurse calves during more than 90 days after calving and are subsequently milked have a longer calving interval but require fewer inseminations to conception compared to cows milked without CCC.

## 2 Animals, materials, and methods

### 2.1 Farm housing and management

From June to September 2023, the study was conducted on an organic commercial farm in Germany. Calf rearing by foster cows was an established farm practice, and no management or housing conditions were changed during the study. The herd consisted of 1,400 Holstein Friesian dairy cows with a mean lactational milk yield of 8,861 kg per lactation. Dairy cows’ feeding consisted of a total mixed ration of corn- and grass-silage, concentrate, minerals, and, depending on lactational stage, straw or linseed- or soy-cake, as well as fresh grass on pasture from April to October for milking cows and fed at the feeding gate for foster cows that had no pasture access.

#### 2.1.1 Foster cows

**Figure 1:**
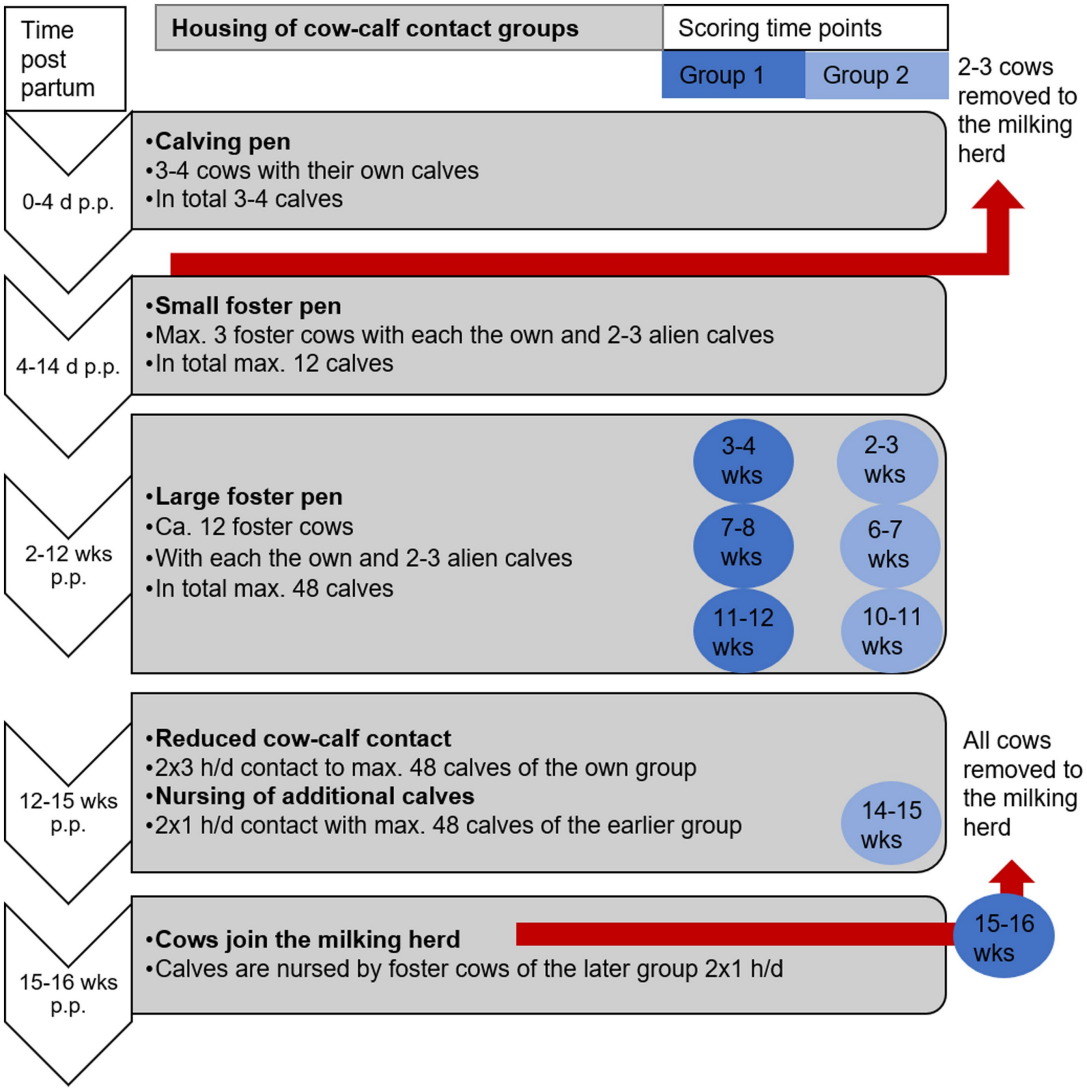
Management of foster cow–calf contact and times of scoring of foster group 1 (dark blue, *n* = 11 cows and 42 calves) and foster group 2 (light blue, *n* = 12 cows and 46 calves) concerning teat condition and body condition (source: Zipp); wks, weeks p.p. (postpartum) when cows were scored.

After calving, all cows and their offspring were housed in a deep litter pen with up to three other dams and their calves. Cows were milked twice daily with a pipeline milking system in the pen. The teats were not dipped. After 3–5 days p.p., one foster cow was chosen for three or four calves. Selection criteria were good health, four lactating teats, preferably multiparity, and tolerant behavior towards calves. Mothers of twins were preferred. After the other two or three cows in the calving pen were transferred to the milking herd, the foster cow and her three to four calves were housed for 2 weeks in a 123-m^2^ deep-litter pen, shared with up to two additional foster cows and their calves (small foster group). Subsequently, the cows and calves were moved to a large 477 m^2^ pen featuring a deep-bedded resting area and an unroofed slatted-floor walking and feeding zone. They were housed in two large foster groups: group 1 (11 cows, 42 calves; 3.8 calves per cow) and group 2 (12 cows, 46 calves; 3.9 calves per cow). Cows in group 1 calved approximately 2 weeks earlier than those in group 2. There was a calf creep, where cows could not enter. Calves were daily locked 2–4 h in the calf creep. Every 2 weeks, the animals were moved as a group to another similar pen in the same building. Gradual weaning included the restriction of full CCC to 3 h twice daily during the 13th to 15th week p.p. During the rest of the day, visual and olfactory contact was possible through fence-line separation. Additionally, for 1 h twice daily, older calves from the earlier foster group—whose foster cows had already been removed—were given access to the foster cows. Starting at the end of the 15th week p.p., foster cows were separated from the calves and transferred to the milking herd. They were milked twice daily until the end of lactation (see Figure 1 for an overview of the management of CCC). During daily health checks, foster cows were restrained in the feeding rack and rewarded with small amounts of concentrate.

#### 2.1.2 Milking cows

Milking cows were separated from their calves 3–5 days p.p. and housed in one of the 16 groups of up to 100 cows in a 1,424-m^2^ barn with deep-bedded cubicles and a solid walking area with automatic scrapers. They were milked twice daily in a side-by-side milking carousel with 66 places (Dairymaster GmbH, Seehausen, DE, MK66, vacuum 48 kPa, common-mode, 68 to 32 pulse cycle). Teats were cleaned automatically with a brush and water. Pre-milking and attachment of milking clusters were performed manually. At a threshold of 200 g/min milk flow, milking clusters were removed automatically. Teats were sprayed automatically after milking with a disinfecting and skin care fluid (Lerapur® Dip MS SP with chlorhexidine and lactic acid, Stockmeier Chemie, Bielefeld, DE). Milking clusters were disinfected after each cow.

### 2.2 Study design and data recording

#### 2.2.1 Selection of study animals

For each of the 23 enrolled foster cows—matched by lactation number and day of calving—a comparable milking cow was selected for the control treatment. The enrolled cows calved between 28 April 2023 and 27 May 2023. Due to unforeseen events, the sample size was reduced to 18 pairs: 2 control cows were slaughtered, 2 foster cows were treated for mastitis and therefore excluded from the study, and 1 foster cow was excluded due to excessive missing data. The mean lactation number for both treatments was 2.7 (±SD 1.5 mean, 1–6 min-max).

#### 2.2.2 Data recording

Teat and body condition were assessed by one trained rater. Inter-rater reliability was evaluated in comparison with the trainer and was found to be good to very good (teat condition: PABAK > 0.90; body condition score: *r* = 0.96; *n* = 34). Teat and body condition were assessed four times. As the cows on the two treatments were scored in their normal housing condition (i.e., milking or foster group), the rater was not blind to the treatment. At the first scoring, cows of the foster group 2 and the paired milking cows were in the 2nd to 3rd week p.p., while cows of foster group 1 and the paired milking cows were in the 3rd to 4th week p.p. There was approximately 1 month between the four scorings (Figure 1). During the first three scorings, all foster cows were in a large foster pen with 20–22 h of calf contact per day. During the fourth scoring, the nine foster cows in group 2 were in the weaning period, with restricted contact to their own 46 foster calves and the 42 foster calves of the earlier foster group. In contrast, the eight foster cows in group 1 (one missing value) had already been separated from their calves 5 days before and had joined the milking herd, where they were milked twice daily (Figure 1). Sample sizes occasionally decreased to 17 or 16 when study cows could not be located in the herd. However, each animal was rated at least three times.

Dryness of teat skin and teat lesions were scored on a 3-point scale according to Elite Magazin (26). Dryness of teat skin was distinguished in (0) smooth skin, (1) slightly dry or rough, and (2) severely dry or rough, skin that is flaking and peeling off. Teat lesions were classified as (0) no lesions, (1) small cracks and lesions, particularly visible at the base of the teat, and (2) significant cracks (horizontally) and evident lesions in the teat.

Body condition score was assessed on a scale from <2 to 4.75 with 0.25 increments [(27, 28); assessment chart by (29)].

The calving interval (days between the calvings in 2023 and 2024) and the number of inseminations to conception were retrieved from farm recordings.

#### 2.2.3 Data processing and statistics

Due to the low frequency of severe teat condition changes, data were dichotomized into 0 = no changes vs. 1 = slight/small or severe/significant changes.

Possible differences in teat lesions and dryness of the teat skin between foster and milked cows were examined using a chi-squared test in R [RStudio 2025.05.0+496, (30)] during each of the four scorings.

The number of overconditioned cows (BCS > 3.75) and the number of underconditioned cows (BCS < 2.5) or cows with rapid BCS changes were also compared between treatments using the chi-squared tests. In the first scoring (2nd to 4th week p.p.), it was checked whether treatments had similar starting points. Across the 2nd to 4th scoring, cows were classified as either overconditioned or underconditioned if they fell into either category at least once. Similarly, rapid body condition changes were rated if cows’ absolute BCS range exceeded 0.5 between the 1st and 4th scoring.

The Mann–Whitney *U*-test [package “coin,” (31)] was used to examine the possible association between teat lesions in foster cows (present at least once during the second and third scorings: yes/no) and their medium BCS during the two scorings. The test was also used to compare calving intervals and numbers of inseminations to conception between foster and milked cows.

The median and median absolute deviation (MAD), as well as the minimum and maximum are given if the Mann–Whitney *U*-tests were used. All tests were two-sided, with a *p*-value of <0.1 interpreted as a tendency and a *p*-value of <0.05 interpreted as a significant difference.

For chi-squared tests, Cohen’s *ω* was calculated as a parameter of effect size with the package “rcompanion” (32). According to Cohen (33), the effect size is interpreted in the following way: *r* = 0.50–1.00 strong effect, *r* = 0.30–0.49 medium effect, *r* = 0.10–0.29 weak effect, *r* = 0.00–0.09, no effect. For the Mann–Whitney *U*-test, the effect size was calculated according to the equation:

$$r=\frac{|Z|}{\surd (n_{\text{foster cows}}+n_{\text{milking cows}})}$$

The effect size *r* can be interpreted in the same way as Cohen’s *ω* (33).

Figures were produced in Microsoft^®^ Excel^®^ (2019 MSO).

## 3 Results

**Figure 2:**
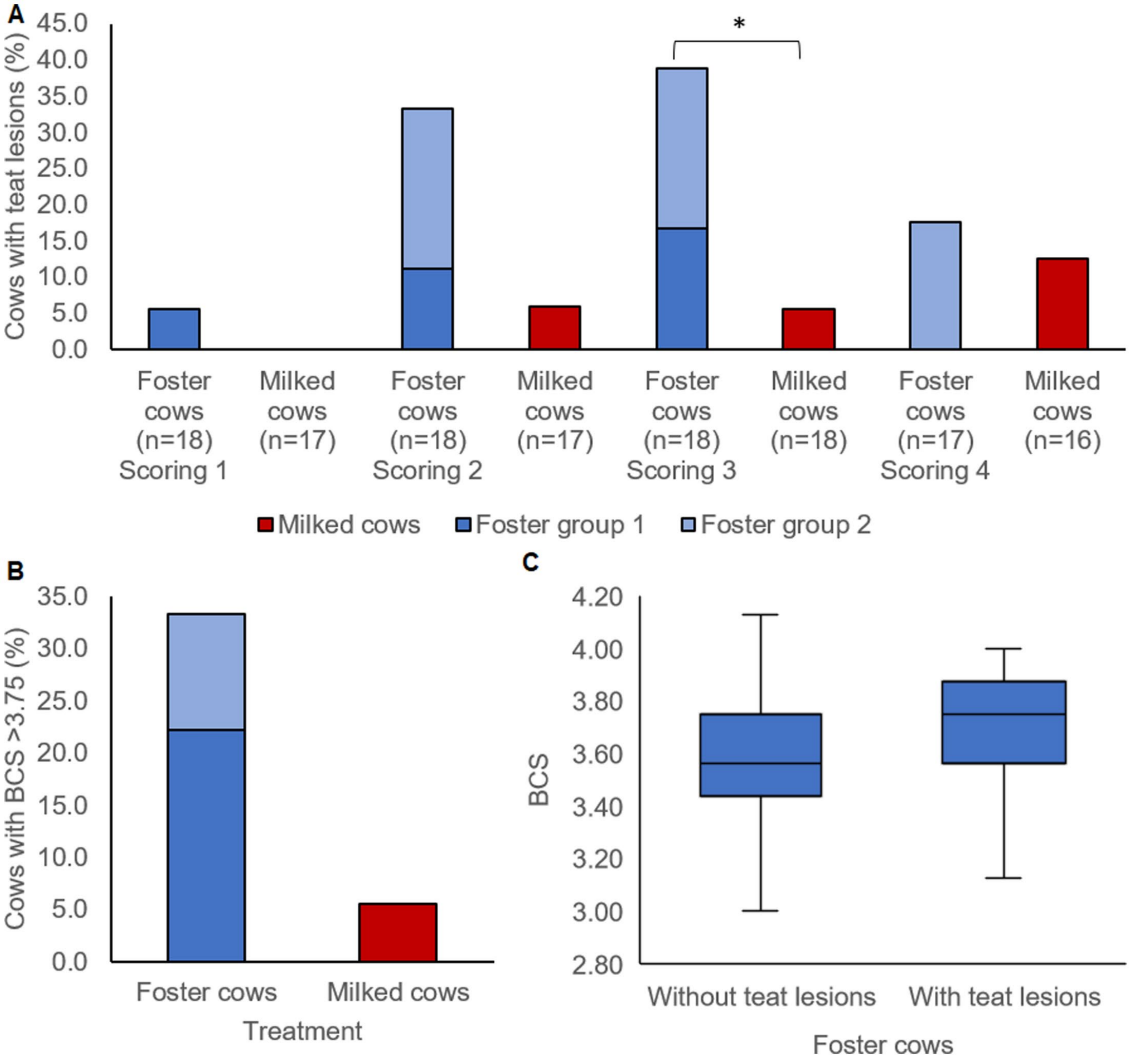
**(A)** Proportion of foster and milked cows with teat lesions during four scorings in the 2nd to 4th week, 6th to 8th week, 10th to 12th week, and 14th to 16th week p.p., star: *p* < 0.05. **(B)** Proportion of cows with body condition score (BCS) > 3.75 in at least one of the three scorings at the 6th to 8th week, 10th to 12th week, and 14th to 16th week p.p. **(C)** Median BCS of 2nd and 3rd scoring of foster cows with teat lesions during at least one of the scorings (*n* = 8) or without teat lesions during both scorings (*n* = 10). Foster cows were housed in two groups with 11–12 cows and their own plus two to three foster calves per cow (*n* = 9 study cows per group). Milked cows were milked twice daily and were housed in a herd of up to 100 dairy cows (*n* = 18 study cows).

The proportion of cows with teat lesions did not differ at the first and fourth scoring between foster and milked cows (Figure 2A, Chi^2^ < 0.01, *p* > 0.1, Cohen’s *ω* < 0.17). During the second scoring (6th to 8th week p.p.), numerically more foster cows had teat lesions with a medium effect (Figure 2A, Chi^2^ = 2.58, *p* = 0.1082, Cohen’s *ω* = 0.34). At the third scoring (10th to 12th week p.p.), this difference was more prominent and significant (Figure 2A, Chi^2^ = 4.02, *p* = 0.04502, Cohen’s ω = 0.40).

Dryness of teat skin did not differ in any scoring between the two treatments (Table 1; results for individual scorings: Supplementary material S1).

**Table 1:** Proportion of foster and milked cows with dry teat skin (range of results on scoring 1–4), and proportion of foster and milked cows that were overconditioned (BCS > 3.75 at scoring 1; scoring 2–4: see Figure 2B), underconditioned (BCS < 2.5), and had rapid BCS changes (absolute range >0.5); scoring 1: 2nd to 4th week p.p., scoring 2: 6th to 8th week p.p., scoring 3: 10th to 12th week p.p., scoring 4: 14th to 16th week p.p.

Parameter	Treatment	*n*	Percentage of cows	Chi^2^	*p*	Cohen’s *ω*
Dry teat skin range from scorings 1 to 4	Foster cows	17–18	0.0%–29.4%	0.00–0.58	>0.1	0.08–0.24
	Milked cows	16–18	12.5%–16.7%			
BCS > 3.75 at scoring 1	Foster cows	17	17.6%	0.00	>0.1	0.00
	Milked cows	18	16.7%			
BCS < 2.5 at scoring 1	Foster cows	17	0.0%	0.00	>0.1	0.17
	Milked cows	18	5.5%			
BCS < 2.5 at any scoring from 2 to 4	Foster cows	18	0.0%	0.53	>0.1	0.24
	Milked cows	18	11.1%			
BCS range > absolute 0.5 between scorings 2 and 3 or 3 and 4	Foster cows	18	11.1%	0.00	>0.1	0.08
	Milked cows	18	16.7%			

At the first scoring, the number of overconditioned or underconditioned foster and milking cows did not differ (Table 1). More foster cows tended to be overconditioned at least once at the second to fourth scoring compared to milked cows (Figure 2B, Chi^2^ = 3.28, *p* = 0.09209, medium effect: Cohen’s *ω* = 0.35). The other BCS parameters did not differ between treatments (Table 1).

With a medium effect size, foster cows with teat lesions had a numerically higher median BCS compared to foster cows without teat lesions during the second and third scoring, but this difference was not statistically significant (Figure 2C, *Z* = −1.27, *p* = 0.2025, *r* = 0.30).

Calving interval and number of inseminations for conception did not differ between treatments (Table 2).

**Table 2:** Calving interval and number of inseminations to conception of foster cows nursing their own and 2–3 foster calves for 3.5 months p.p. and being milked until the end of lactation, and cows being milked only twice daily.

Parameter	Treatment	*n*	Median	MAD	Min	Max	*Z*	*p*	*r*
Calving interval (days)	Foster cows	13	412	13	386	626	0.63	0.5315	0.13
	Milked cows	12	421	41	319	547			
Number of inseminations	Foster cows	13	1	0	1	5	−1.09	0.2737	0.22
	Milked cows	12	2	1	1	6			

## 4 Discussion

This study evaluated welfare risks in high-yielding foster cows nursing 3–4 calves—both their own and foster—for approximately 3.5 months before returning to the milking herd. We compared their teat and body condition, as well as fertility, with those of milked-only cows.

Key findings showed a significantly higher prevalence of teat lesions in foster cows between the 10th and 12th weeks of nursing, although lesions were predominantly small and decreased until the final scoring. Foster cows also showed a trend toward overconditioning. No significant differences were found in body condition, fertility measures, or teat dryness. However, the results suggest that there may be aversive dynamics involved in foster cow–calf interactions.

Even though teat lesions were primarily small and had healed in the eight cows that had been separated from their calves 5 days earlier, they still represent a welfare concern. Teat lesions are potentially painful and increase the risk of infection (34). The finding of an increased risk of teat lesions in foster cows is in line with other studies (13, 14, 35). Since foster group size and composition remained constant over the first three scorings, the increase in teat lesions from the 2nd to 4th week to the 10th to 12th week of lactation is likely due to behavioral changes. Calves may have suckled more vigorously or increased allosuckling from specific cows that were more calf-tolerant. This may have led to competition between calves. In fact, on the same farm, Wieczorreck and Hillmann (36) observed that, with increasing age, foster calves increasingly performed allosuckling. Conversely, reduced suckling tolerance of cows may have led to increased biting behavior in calves, as we found a trend associating teat lesions with higher BCS (medium effect).

If aversive interactions occur between foster cows and calves, this raises welfare concerns. One underlying issue may be the lack of cow–calf bonding in foster systems. Grouping foster cows with their foster calves as soon as possible after their parturition may enhance calf acceptance (37, 38). In the meantime, the farm has indeed reduced the delay between birth and grouping from 3 to 5 days to 24 h. Improved calf acceptance can also be achieved by odor transfer from the own cow to the foster calf using cloth jackets. This method has been demonstrated to be effective in beef suckler systems (39), but feasibility may be low. Further studies should investigate the effects of such strategies on cow–calf acceptance in foster systems.

In the group of foster cows that still suckled the highest number of calves (a total of 88 calves for 12 cows), teat lesion prevalence also declined. This suggests increased calf independence from milk, although under semi-natural conditions, calves are typically weaned by the dam only at an age of 6–10 months (40). Earlier calf independence is also reflected in findings from the current farm (41), as calf weight gain did not decline during the weaning process at approximately 15 weeks of age. Similarly, Ivemeyer et al. (42) observed reduced milk intake of calves with 11-15 weeks of age during gradual weaning with foster cow contact in a mixed dam–foster CCC system with twice-daily contact, suggesting increased ingestion of solid feed.

In cases where teat lesions are due to calf hunger, Barth et al. (17) recommended reducing the number of calves per cow. However, this may increase BCS, as foster cows may produce more milk than the calves would ingest. Given the observed tendency toward overconditioning in foster cows, this strategy may lead to even more overconditioned cows, with an increased risk of associated health impairments (43). Moreover, a further unintended consequence could be a reduction of machine-milk yield after separation, as lower udder emptying frequency during early lactation diminishes milk synthesis and influences the entire lactation curve [reviewed by (44)]. A decreasing effect on lactational milk yield has been observed in some dam-rearing studies, particularly with whole-day contact [e.g., (21, 25, 45, 46)].

The results did not confirm our hypothesis that foster cows in general are more underconditioned (BCS < 2.5) or have more rapid BCS changes. Furthermore, Thomas et al. (13) did not find an effect of multiple suckling vs. twice daily milking on BCS, but other studies found a decreasing BCS with increasing frequency of milk removal (19–21). Although foster cows in the current study showed a trend toward a higher BCS, overconditioning occurred in both treatments, as well as rapid changes in body condition. This should be avoided for health reasons (29). Underconditioning occurred in some milked cows. Therefore, the farm should adjust feeding more closely to body condition and lactation stage.

Even though foster cows had CCC for 3.5 months and cow–calf bonding can lead to delayed estrus [reviewed by (23)], calving interval did not differ between treatments, which confirms the findings of Zipp and Knierim (25) on another farm with dam CC for 9 weeks. The number of inseminations also did not differ between treatments. However, it should be considered that the median calving interval for both treatments was relatively long, with more than 400 days, and foster cows were only inseminated after separation from the calves.

As a limitation of the study, blinding the rater was not possible, which carries the risk of expectation bias. At least during the last scoring, the results were against our expectations. However, for future studies, blinding of the rater would be advisable. Furthermore, it should be considered that some housing and management conditions, which may have influenced the outcomes, were not balanced between treatments, e.g., cubicle housing plus pasture access vs. deep litter plus unroofed area, and grazing vs. feeding fresh grass. Wieczorreck et al. (47) observed on the same farm lower lying durations of milking vs. foster cows, possibly due to pasture access and the milking procedure, which may all have affected the body condition. Cows with clinical mastitis were not included in the study. However, considering farm-specific problems with *Pasteurella* spp. in the foster group [see (16)], further investigations of associations between teat lesions and udder health would be worthwhile. In general, it must be considered that CCC systems are very heterogenous, which limits the generalizability of the conclusions from this study.

In conclusion, while foster cow–calf systems offer a more natural rearing approach, we found a higher risk of teat lesions. Teat lesions, however, did not increase during and after the weaning period. Teat lesions are likely not only painful and may promote infections but may also partly indicate aversive relationships between foster cows and calves. This is altogether a welfare concern. No significant effects on the dryness of teat skin, body condition, or fertility outcomes were detected. However, a trend toward more overconditioned cows and, particularly, a numerically higher BCS in foster cows with teat lesions (medium effect size) suggests the need for larger-scale studies to investigate the underlying causes of teat lesions in relation to foster cows’ tolerance of suckling.

## Data Availability

The raw data supporting the conclusions of this article will be made available by the authors, without undue reservation.
