# Supplementary material for: Brief research report: Fertility, teat, and body condition of foster cows in a cow–calf contact system
Source: Front Vet Sci. 2026 Jan 21;12:1678081. doi: 10.3389/fvets.2025.1678081 (PMC12870695; doi:10.3389/fvets.2025.1678081)
Supplement: Supplementary file 1 [file Table_1.docx]

Supplementary Material 1: Proportion of foster and milked cows with dry teat skin, scoring 1: 2^nd^ to 4^th^ wk p.p., scoring 2: 6^th^ to 8^th^ wk p.p., scoring 3: 10^th^ to 12^th^ wk p.p., scoring 4: 14^th^ to 16^th^ wk p.p.. Foster cows were housed in two groups with 11-12 cows and their own plus two to three alien calves per cow (n=9 study cows per group). Milked cows were milked twice daily and were housed in a herd of up to 100 dairy cows (n=18 study cows). Sample sizes are sometimes reduced to 17 or 16 when study cows could not be found in the herd for scoring.

| Scoring | Treatment | n | percentage of cows | Chi² | p | Cohen’s ω |
| --- | --- | --- | --- | --- | --- | --- |
| 1 | foster cows | 18 | 11.1% | <0.01 | >0.1 | 0.08 |
|  | milked cows | 17 | 17.6% |  |  |  |
| 2 | foster cows | 18 | 22.2% | 0.14 | >0.1 | 0.14 |
|  | milked cows | 17 | 11.8% |  |  |  |
| 3 | foster cows | 18 | 0.0% | 0.53 | >0.1 | 0.24 |
|  | milked cows | 18 | 11.8% |  |  |  |
| 4 | foster cows | 17 | 29.4% | 0.58 | >0.1 | 0.20 |
|  | milked cows | 16 | 12.5% |  |  |  |
